# Selective Penetration and Profile Control Performance of Preformed Particle Gels for Heterogeneous Oil Reservoirs

**DOI:** 10.3390/gels8100601

**Published:** 2022-09-21

**Authors:** Kang Zhou, Dejun Wu, Zhibin An, Shuai Liu

**Affiliations:** 1 College of Energy and Mining Engineering, Shandong University of Science and Technology, Qingdao 266590, China; 2 School of Petroleum Engineering, China University of Petroleum (East China), Qingdao 266580, China

**Keywords:** preformed particle gel, heterogeneous oil reservoir, selective penetration, profile control ability

## Abstract

The preformed particle gel (PPG) has been proved to be an effective chemical agent to reduce fluid channeling and increase the sweeping efficiency. However, we still lack a clear understanding of the field-scale matching relationship between PPG size, elastic modulus and a heterogeneous reservoir. In this respect, the paper carried out various sand pack displacement experiments. The results indicated that an excessively large PPG or elastic modulus would plug a low-permeability sand pack and even increase the severity of fluid channeling. On the contrary, an excessively small PPG or elastic modulus allowed a certain degree of profile control, but the PPG could easily migrate out of high-permeability sand packs with water. If the elastic modulus remained unchanged, the suitable PPG size increased as the reservoir permeability ratio increased. On the other hand, the suitable elastic modulus increased with the increase of the reservoir permeability ratio when the PPG size was kept the same. By using regression analysis, quantitative expressions were established in order to determine the best suitable PPG size for a certain heterogeneous reservoir. When the elastic modulus was fixed, the best suitable PPG mesh exhibited a linear relation with the permeability ratio. This paper provides a useful reference to select the most convenient PPG size and elastic modulus for a potential heterogeneous reservoir, suitable to enhance oil recovery.

## 1. Introduction

Most water-flooding oilfields in China can only achieve an oil recovery of about 30%, which can be further increased to no more than 50% through polymer flooding after water flooding. In order to further increase the sweeping efficiency and improve oil recovery in strong heterogeneous reservoirs, the preformed particle gel (PPG) has been developed and proved to be a promising fluid-diverting agent. Due to its capacity of blockage, deformation and re-migration, PPG can be transported into a deep reservoir and block the fluid channeling paths, driving the displacing fluid to the unswept regions of the reservoir that contain rich crude oil and finally improving oil recovery. Because of its promising application prospects, researchers have carried out many studies on the preparation, migration and oil-displacing characteristics of PPG.

As for the preparation and perfection of particle gels, Bai et al. [1] studied the effect of the gelant composition, temperature, brine salinity, and pH on the swollen gel strength and swelling capacity of PPG. Moghadam et al. [2] applied a statistical technique to establish a functional relationship between salinity, pH and equilibrium swelling ratio. Dai et al. [3] described a simple high-speed shearing method to obtain the desired dispersed particle gel. Yang et al. [4] carried out studies on the influencing factors of initial particle size and swelling capability. Imqam et al. [5] optimized the strength and size of PPG in order to achieve a better profile control performance. Saghafi et al. [6] focused on improving the thermo-chemical stability of PPG by incorporating a new monomer in well-known nano-composite PPGs. Khoshkar et al. [7] analyzed the effect of nanomaterials on enhanced oil recovery by a novel synthesized PPG in fractured reservoirs.

The transport of PPG in porous media has been another research focus in recent years. Bai et al. [8] studied PPG propagation and water flow through open fractures and discussed the effect of injection rate, fracture width and swelling ratio. Wang et al. [9] established a flow model of PPG in porous media. Elsharafi et al. [10] investigated the influence of particle size, brine concentration and gel strength on PPG blocking efficiency. By using experimental methods, Song et al. [11] studied the injection characteristics, Imqam et al. [12] studied the plugging performance, Saghafi et al. [13] studied the retention characteristics, and Li et al. [14] studied the seepage and displacement characteristics of PPG in conformance control. Zhao et al. [15] investigated the restarting pressure gradient of PPG to deform and pass through pore throats. Zhou et al. [16] further studied the flow resistance of the dispersion system of PPG, which is important for the injection of PPG suspensions into porous media.

Since PPG was mainly used to decrease the water cut and improve oil recovery, researchers have conducted various studies on the displacement performance of PPG. Sang et al. [17] studied the flow diversion and increasing sweeping efficiency resulting from the injection of PPG using parallel sand pack models. Goudarzi et al. [18] also focused on the conformance control performance of PPG. Gong et al. [19] analyzed the synergistic effect of PPG and a hydrolyzed polyacrylamide to enhance oil recovery in reservoirs after polymer flooding. Zhao et al. [20] analyzed the combination of a dispersed particle gel and a polymer/surfactant to further enhance oil recovery. Sun et al. [21] discussed the combination of PPG and low-salinity water flooding in fractured reservoirs. Wu et al. [22] carried out an experimental study on combining PPG/polymer/surfactant and streamline adjustment to improve the oil recovery in heterogeneous reservoirs. As for field applications, Zhao et al. [23] and Dai et al. [24] also studied the matching relationship between particle gels and oil reservoirs. Wang et al. [25] and Bai et al. [26] discussed some limitations of the current research, which should be considered in the studies on matching relationships.

Although some studies were performed on PPG injection, as reviewed above, the field-scale matching relationship between PPG size, elastic modulus and reservoir heterogeneity has not been studied. The existing research works simply studied the profile improvement ability and increased oil recovery performance of a PPG sample with a certain particle size and elastic modulus. In contrast, this paper aimed at finding the best suitable PPG for a heterogeneous reservoir by performing system experiments. In the paper, the experimental methods including materials, apparatus, procedure and evaluation criteria are first detailed. Then, the relationship between PPG properties and reservoir permeability ratio is analyzed. In addition, the quantitative expressions describing the matching relationship were developed by using regression analysis.

## 2. Materials and Methods

### 2.1. Materials

The PPG dry powder used in the displacement experiments was provided by Shengli Oilfield. These PPG agents had two values of elastic modulus, i.e., 2–4 Pa and 12–16 Pa. In order to study the effect of the PPG size on the profile control performance, the dry powder was divided into several groups according to its particle size distribution, i.e., 20–40 mesh, 40–60 mesh, 60–80 mesh, 80–100 mesh, 100–120 mesh, 120–150 mesh and 150–180 mesh. It needs to be emphasized that the elastic modulus and particle size mentioned in this paper refer to the dry powder state. In this study, the PPG was injected as a particle suspension with the constant mass concentration of 2000 mg/L.

Parallel sand pack models were used in the displacement experiments. The diameter of each sand pack model was 25 mm, and their length was 300 mm. Before every displacement experiment, the sand pack model was prepared by filling with suitable quartz sands. In fact, different sand sizes and compaction degrees were tried in order to obtain parallel sand packs with the expected permeability ratios.

### 2.2. Experimental Apparatus

**Figure 1:**
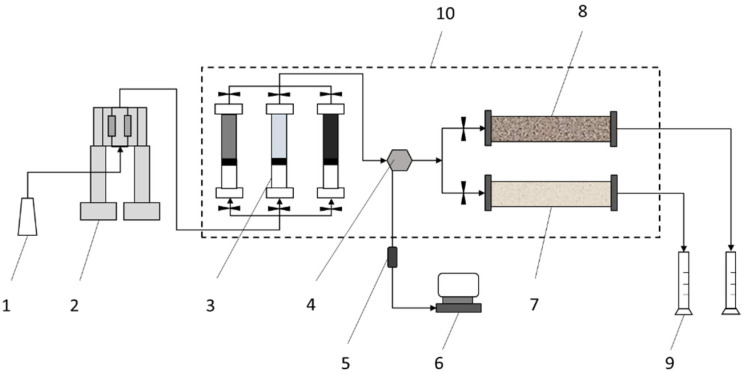
Schematic of the sand pack model displacement apparatus. 1, distilled water; 2, ISCO pump; 3, intermediate container; 4, six-way valve; 5, pressure sensor; 6, pressure acquisition system; 7, low-permeability sand pack model; 8, high-permeability sand pack model; 9, measuring cylinder; 10, temperature control box.

The displacement experimental apparatus was set up as shown in Figure 1. As can be seen, the displacement system mainly consisted of an ISCO pump, an intermediate container with water, a PPG suspension, an oil pressure sensor and acquisition system, a parallel sand pack model, a temperature control box and a measuring cylinder to collect the produced oil and water. During the experiments, the injection rate of the ISCO pump was set to 1.0 mL/min. The temperature control box was set to 70 °C.

### 2.3. Experimental Procedure

In the displacement experiments, three slugs were successively injected, which consisted of water, a PPG suspension, and water. The main experimental procedure was as follows:

1. Prepare the parallel model and measure the porosity and permeability of each sand pack;
2. Displace the parallel model successively using about 1 PV of water, 0.6 PV of PPG suspension and, again, 1.4 PV of water;
3. Record the injection pressure every 1 min and measure the liquid flux of each sand pack every 10 min;
4. Analyze the experimental data and establish the matching relationship between the PPG properties and reservoir heterogeneity.

### 2.4. Evaluation Criteria

The profile control ability of the PPG is the key to successfully changing the water flow direction and increasing the sweeping efficiency. In fact, field applications should pay more attention to the increase of the fractional flow in low-permeability regions. Therefore, the evaluation criterion was defined as the variation degree of the fractional flow of the low-permeability sand pack before and after PPG injection [27]. It is described by Equation (1).

(1) $$f=\frac{f_{la}-f_{lb}}{f_{lb}}=\frac{\frac{Q_{la}}{Q_{la}+Q_{ha}}-\frac{Q_{lb}}{Q_{lb}+Q_{hb}}}{\frac{Q_{lb}}{Q_{lb}+Q_{hb}}}$$

where $f_{la}$ indicates the fractional flow of the low-permeability sand pack after PPG injection; $f_{lb}$ indicates the fractional flow of the low-permeability sand pack before PPG injection; $Q_{hb}$ indicates the flux in the high-permeability sand pack before PPG injection; $Q_{ha}$ indicates the flux in the high-permeability sand pack after PPG injection; $Q_{lb}$ indicates the flux in the low-permeability sand pack before PPG injection; $Q_{la}$ indicates the flux in the low-permeability sand pack after PPG injection.

## 3. Results and Discussion

**Figure 2:**
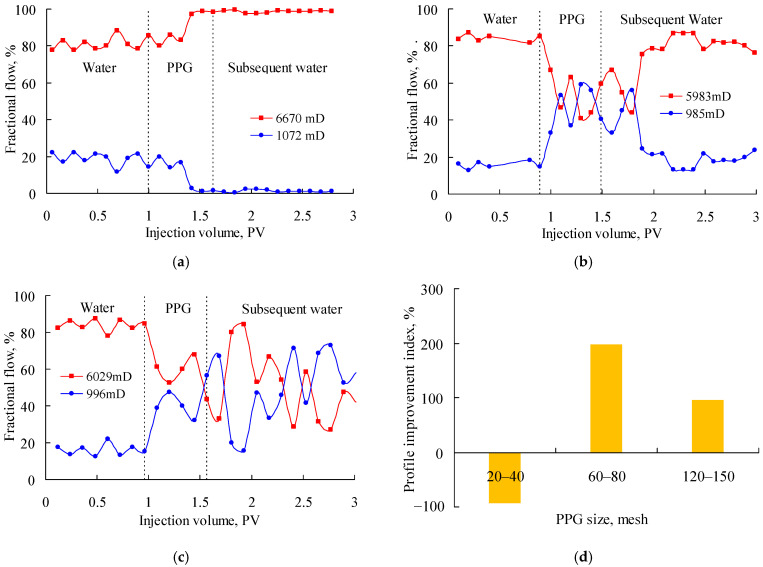
Fractional flow of parallel sand packs with permeability ratio of about 6 after injecting the PPG (2–4 Pa). (**a**) 20–40 mesh; (**b**) 120–150 mesh; (**c**) 60–80 mesh; (**d**) profile improvement index.

Figure 2 shows the fractional flow when PPG (2–4 Pa) suspensions with particle sizes of 20–40 mesh, 60–80 mesh and 120–150 mesh were injected into the parallel sand packs with permeability ratio of about 6. In the Figure, the red solid lines indicate the fractional flow in the high-permeability sand pack, the blue solid lines represent the fractional flow in the low-permeability sand pack, and the black broken lines represent the stage division of water flooding, PPG flooding and subsequent flooding. As can be seen, the fractional flow in both sand packs was relatively stable in the water injection stage. However, after injecting the PPG suspension, the fractional flow in each sand pack began to change in different ways. As shown in Figure 2a, during the injection of the 20–40-mesh PPG, the fractional flow increased in the high-permeability sand pack and decreased in the low-permeability sand pack. Gradually, the ratio of the fractional flow in the two sand packs was close to 100:0. This was mainly because the 20–40-mesh PPG was excessively large, and, as a result, that the low-permeability sand pack was plugged. Therefore, the production in the low-permeability sand pack dropped significantly. At the same time, since the total flow rates of the two parallel sand packs were constant, at 0.1 mL/min, and remained unchanged throughout the experiments, the production in the high-permeability sand pack increased accordingly. As shown in Figure 2b, during the injection of the 120–150-mesh PPG, the fractional flow decreased rapidly in the high-permeability sand pack, fluctuating around 50%, which indicated a good profile control performance. However, during the injection of subsequent water, the fractional flow increased again in the high-permeability sand pack. This was mainly because the 120–150-mesh PPG was excessively small for the high-permeability sand pack and was easily carried out by the subsequent water. As shown in Figure 2c, during the injection of the 60–80-mesh PPG, the fractional flow decreased in the high-permeability sand pack, and then the fractional flow fluctuated alternately in the two sand packs. Even in the subsequent water injection stage, the fractional flow in the low-permeability sand pack remained high. This indicated the 60–80-mesh PPG had a good profile control ability during the injection of the PPG suspension and then of water. According to the changes of the fractional flow before and after the PPG injection, the profile improvement index was calculated, as shown in Figure 2d. The profile improvement index of the PPG at 2–4 Pa, 60–80 mesh, was the highest and reached 209.86%; therefore, this PPG best matched the heterogeneous sand packs with a permeability ratio of about 6.

**Figure 3:**
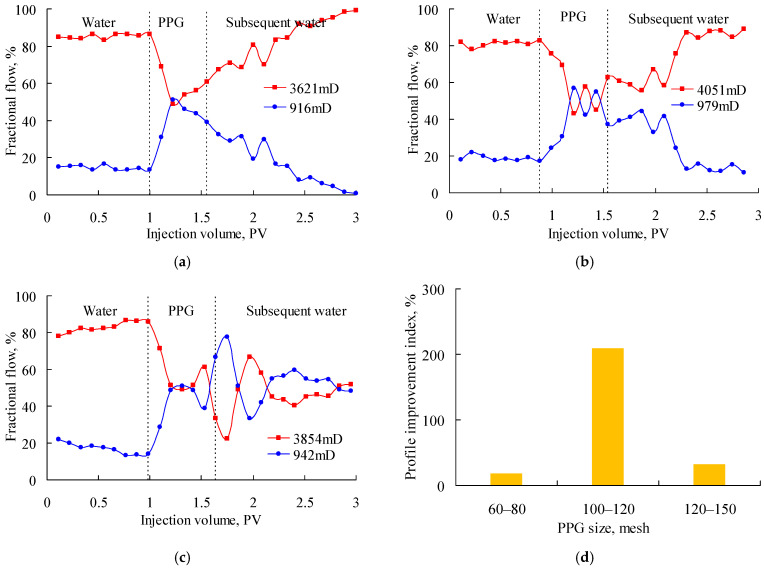
Fractional flow of parallel sand packs with permeability ratio of about 4 after injecting the PPG (2–4 Pa). (**a**) 60–80 mesh; (**b**) 120–150 mesh; (**c**) 100–120 mesh; (**d**) profile improvement index.

Figure 3 shows the fractional flow when the PPG (2–4 Pa) suspensions with particle sizes of 60–80 mesh, 100–120 mesh and 120–150 mesh were injected into the parallel sand packs with a permeability ratio of about 4. As shown in Figure 3a, the fractional flow decreased largely in the high-permeability sand pack after the PPG injection but increased again in the subsequent water injection stage and finally reached about 100%. It indicated that the 60–80-mesh PPG was excessively large and gradually plugged the low-permeability sand pack. As shown in Figure 3b, the fractional flow in the high-permeability sand pack decreased rapidly during the PPG injection but increased again after the subsequent water injection. It indicated that the 120–150-mesh PPG was excessively small. As shown in Figure 3c, in the processes of both PPG injection and subsequent water injection, the fractional flow of the two sand packs fluctuated alternately around 50%, reflecting a good profile control performance. Figure 3d also shows that the 100–120-mesh PPG obtained the highest profile improvement index of 209.56%; therefore, it best matched with sand packs with a permeability ratio of about 4.

**Figure 4:**
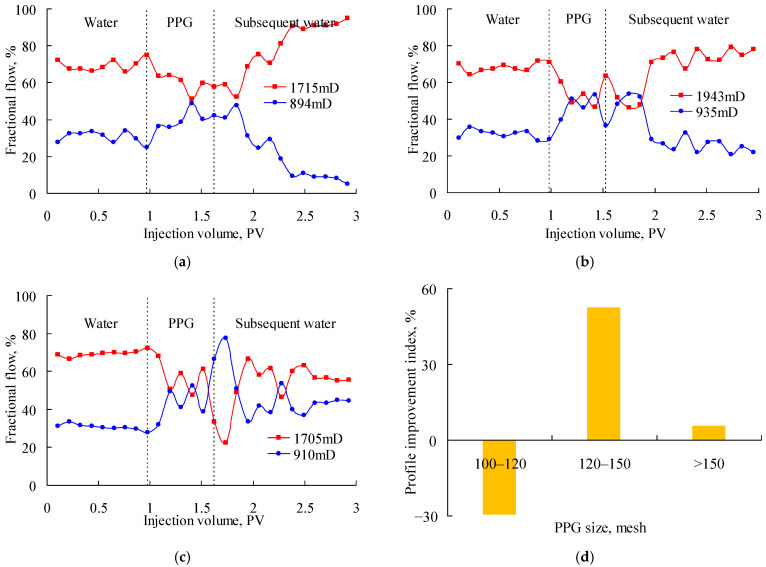
Fractional flow of parallel sand packs with permeability ratio of about 2 after injecting the PPG (2–4 Pa). (**a**) 100–120 mesh; (**b**) 150–180 mesh; (**c**) 120–150 mesh; (**d**) profile improvement index.

Figure 4 shows the fractional flow when PPG (2–4 Pa) suspensions with particle sizes of 100–120 mesh, 120–150 mesh and 150–180 mesh were injected into the parallel sand packs with a permeability ratio of about 2. As shown in Figure 4a, the fractional flow in the high-permeability sand pack decreased slightly during the PPG injection and the early stage of the subsequent water injection. However, it increased almost to 100% at the end of the experiment, which indicated the low-permeability sand-pack was plugged due to the excessively large size of the 100–120-mesh PPG. As shown in Figure 4b, the fractional flow in the two sand packs fluctuated around 50% during the late stage of the PPG injection and the early stage of the subsequent water injection. However, its profile control performance decreased in the late displacement stage, which indicated that the 120–150-mesh PPG was excessively small. As shown in Figure 4c, the fractional flow in the two sand packs continuously improved after the injection of the PPG suspension. Figure 4d also shows that the 100–120-mesh PPG obtained the highest profile improvement index of 52.71%; therefore, it best matched with the sand packs with a permeability ratio of about 2.

**Figure 5:**
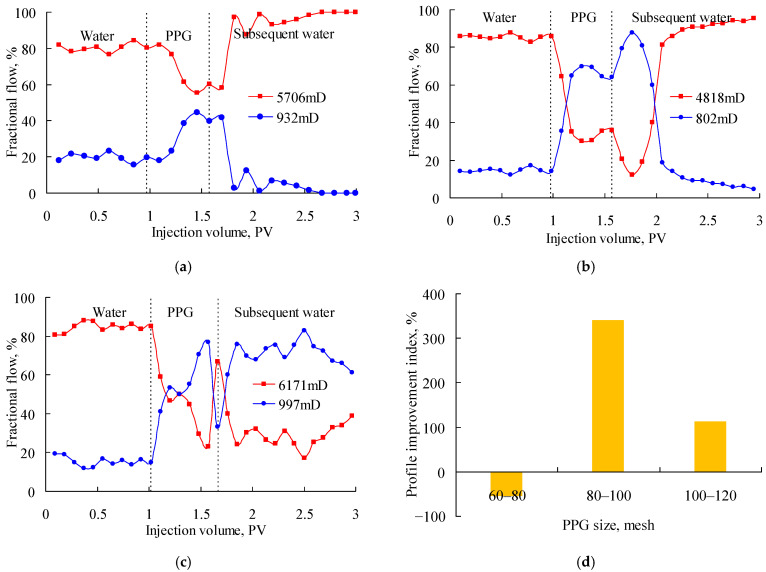
Fractional flow of parallel sand packs with permeability ratio of about 6 after injecting the PPG (12–16 Pa). (**a**) 60–80 mesh; (**b**) 100–120 mesh; (**c**) 80–100 mesh; (**d**) profile improvement index.

Figure 5 shows the fractional flow when the PPG (12–16 Pa) suspensions with particle sizes of 60–80 mesh, 80–100 mesh and 100–120 mesh were injected into parallel sand packs with the a permeability ratio of about 6. As shown in Figure 5a, the fractional flow in the high-permeability sand pack decreased slightly during the PPG injection but increased soon after the subsequent water injection. Because of the excessive size of the 60–80-mesh PPG, the low-permeability sand pack was blocked, and its fractional flow became even smaller than the original value. As shown in Figure 5b, the fractional flow decreased largely in the stage of PPG injection and in the early stage of the subsequent water injection. Since the 100–120-mesh PPG was excessively small, the profile control performance was null when the PPG migrated out of the sand pack. Figure 5c shows that the fractional flow in the low-permeability sand pack increased largely and remained at a high level until the end of the experiment. Its profile improvement index was 340.71%, as shown in Figure 5d. Therefore, the 80–100-mesh PPG best matched with the sand packs with a permeability ratio of about 6.

**Figure 6:**
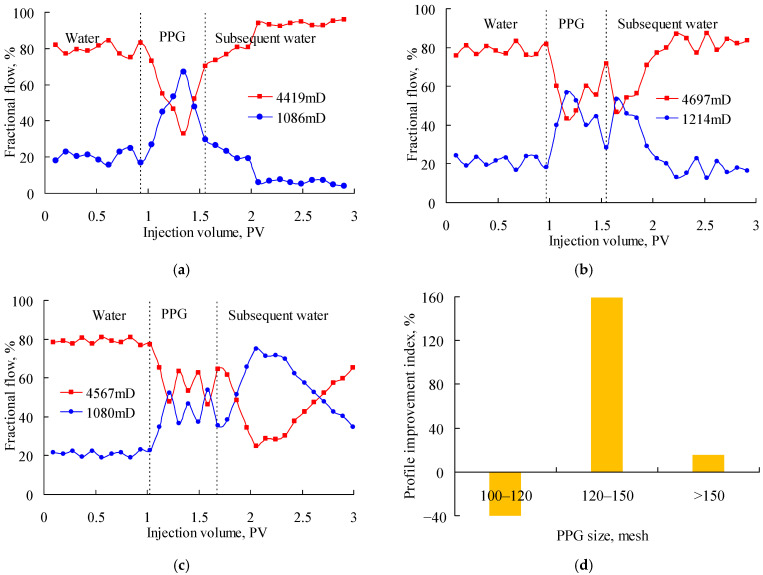
Fractional flow of parallel sand packs with permeability ratio of about 4 after injecting the PPG (12–16 Pa). (**a**) 100–120 mesh; (**b**) 150–180 mesh; (**c**) 120–150 mesh; (**d**) profile improvement index.

Figure 6 shows the fractional flow when PPG (12–16 Pa) suspensions with particle sizes of 100–120 mesh, 120–150 mesh and 150–180 mesh were injected into parallel sand packs with a permeability ratio of about 4. As shown in Figure 6a, the fractional flow in the high-permeability sand pack decreased largely after injecting the PPG but then increased and even became larger than the original flow. This indicated that the 100–120-mesh PPG was excessively large. As shown in Figure 6b, the fractional flow in the two sand packs fluctuated around 50% during the PPG injection. However, the profile improvement performance became null soon after the injection of water, showing that the 150–180-mesh PPG was excessively small. Figure 6c shows that the 120–150-mesh PPG performed well because the fractional flow in the low-permeability sand pack increased largely and remained at a satisfactory level. As shown in Figure 6d, the profile improvement index of the 120–150-mesh PPG was 159.48%. Therefore, the 120–150-mesh PPG best matched with the sand packs with a permeability ratio of about 4.

**Figure 7:**
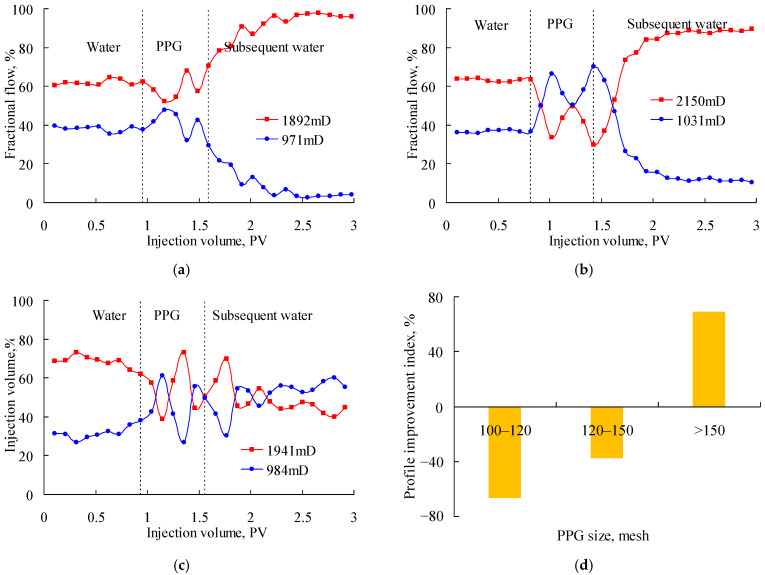
Fractional flow of parallel sand packs with permeability ratio of about 2 after injecting the PPG (12–16 Pa). (**a**) 100–120 mesh; (**b**) 120–150 mesh; (**c**) 150–180 mesh; (**d**) profile improvement index.

Figure 7 shows the fractional flow when PPG (12–16 Pa) suspensions with particle sizes of 100–120 mesh, 120–150 mesh and 150–180 mesh were injected into parallel sand packs with a permeability ratio of about 2. As shown in Figure 7a, the fractional flow in the high-permeability sand pack decreased a little after injecting the PPG and soon increased rapidly to a large value, indicating that even a small quantity of 100–120-mesh PPG could block the low-permeability sand pack. As shown in Figure 7b, the 120–150-mesh PPG obtained a good profile improvement performance in the stage of PPG injection. However, the fractional flow in the low-permeability sand pack decreased rapidly after injecting the subsequent water because the PPG retention increased the flow resistance in the low-permeability sand-pack. When the PPG size decreased further to 150–180 mesh as shown in Figure 7c, the fractional flow in the two sand packs fluctuated around 50% until the end of the displacement experiments. Figure 7d also shows the negative profile improvement index for the 100–120-mesh and 120–150-mesh PPG, indicating that the excessively large PPGs plugged the low-permeability sand pack and resulted in a severer fluid channeling in the high-permeability sand pack. Therefore, the 150–180-mesh PPG best matched with the sand packs with a permeability ratio of about 2.

**Figure 8:**
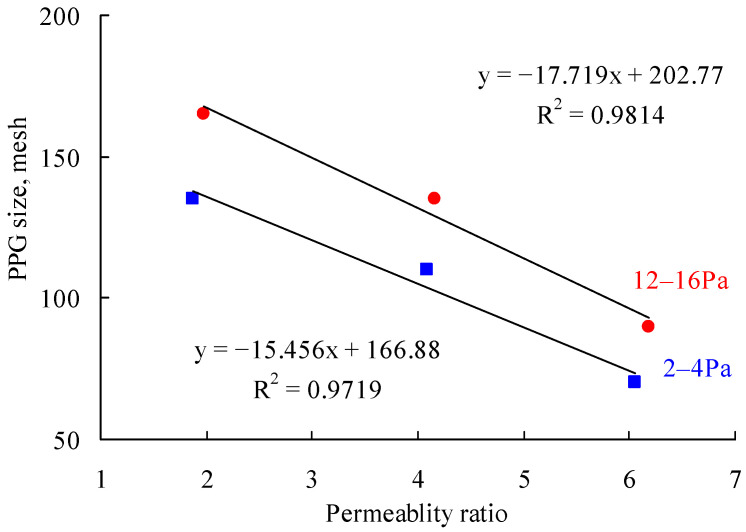
Matching relationship between PPG size, elastic modulus and permeability ratio.

Figure 2, Figure 3, Figure 4, Figure 5, Figure 6 and Figure 7 compare the variation of the fractional flow after injecting PPG suspensions with different sizes and elastic modulus into parallel sand packs with different permeability ratios. The matching relationship curves between the PPGs and the permeability ratios were obtained accordingly, as shown in Figure 8. As can be seen, for a certain value of the elastic modulus of the PPG, the matching mesh of the PPG decreased as the permeability ratio increased. When the PPG mesh remained unchanged, the matching elastic modulus of the PPG increased with the increase of the permeability ratio. In other words, the larger the permeability ratio, the larger the PPG size and the elastic modulus. By using regression analysis, the matching PPG mesh and permeability ratio exhibited a linear relation.

## 4. Conclusions and Limitations

From the experimental study, some conclusions were obtained:

1. An excessively large PPG or elastic modulus may plug a low-permeability sand pack and even result in a very severe fluid channeling. An excessively small PPG or elastic modulus had a certain degree of profile control, but the PPG could easily migrate out of high-permeability sand packs with subsequent water.
2. The best suitable PPG size and elastic modulus increased with the increase of the reservoir permeability ratio. When the elastic modulus was fixed, the best suitable PPG mesh exhibited a linear relationship with the permeability ratio.

This research has some limitations. The conclusions were obtained from experiments carried out using short sand pack models, with a length of 30 cm, supposing that the PPG can propagate deep into a reservoir. However, it is not very clear how deep a PPG can propagate and what effects it may cause on real reservoirs. In addition, this research was based on a specific PPG product, as mentioned in the Material section. In fact, various similar products have been studied by many researchers; therefore, the matching relationship needs further development before it is possible to apply it in oilfields. However, we think the experimental methods here described are always effective.

## Data Availability

Data available on request from the authors.
